# Modeling the Activated Sludge—Thickening Process in Secondary Settlers

**DOI:** 10.3390/ijerph121214996

**Published:** 2015-12-04

**Authors:** Yuankai Zhang, Xunfei Yin, Zhijiang He, Xiangjun Zhang, Yang Wen, Hongchen Wang

**Affiliations:** School of Environment & Natural Resource, Renmin University of China, Beijing 100872, China; zyk11@ruc.edu.cn (Y.K.); yinxunfei2009@163.com (X.F.); hezhijiang@ruc.edu.cn (Z.J.); zxj@ruc.edu.cn (X.J.); wenyanggrf@126.com (Y.W.)

**Keywords:** secondary settlers, sludge settling, sludge thickening, sludge volume index, compression zone

## Abstract

A single paragraph of about 200 words maximum. For research articles; abstracts should give a pertinent overview of the work. We strongly encourage authors to use the following style of structured abstracts; but without headings: (1) Background: Place the question addressed in a broad context and highlight the purpose of the study; (2) Methods: Describe briefly the main methods or treatments applied; (3) Results: Summarize the article's main findings; and (4) Conclusion: Indicate the main conclusions or interpretations. The abstract should be an objective representation of the article: it must not contain results which are not presented and substantiated in the main text and should not exaggerate the main conclusions.

## 1. Introduction

Wastewater without proper treatment causes a serious threat to the water environment. Currently, over 50% of surface water in the seven main rivers in China have been seriously polluted by wastewater, requiring adequate treatment of wastewater discharging to the rivers. In December 2014, there were 3717 municipal wastewater treatment plants (WWTPs) under operation in China, with a total capacity of 1.57 × 10^8^ m^3^/d. About 5.1 × 10^7^ kWh/d of energy were consumed by WWTPs, largely due to the inadequate optimization of process operations [1]. Secondary settlers are often the great challenge in the activated sludge process operation, and are crucial for the overall performance and efficiency of wastewater treatment [2]. Many models had been developed to describe the activated sludge settling and thickening process, including one-dimensional [3], two-dimensional [4], and three-dimensional [5] models, which were useful for optimizing the activated sludge systems. Among the models, one-dimensional models are used most frequently, because they do not require substantial computational efforts or careful estimations of multiple parameters [6].For example, in the famous Kynch theory[7], the concept of solid flux was easily adapted in the design and operation of WWTPs.

The sludge settling velocity is an important parameter for evaluating the total solids flux in a secondary settler. Generally it includes two steps: (1) settling at low concentrations (including discrete and flocculent particle settling), and (2) settling at high concentrations (including sludge hindered and compression settling) [8]. In low concentration settling, the isolated floc settling velocity was found to be dependent on their sizes [9]. For high concentration settling, the velocity is also correlated with sludge concentrations, as shown by the well-known Vesilind equation and Takács model [8,10]. However, current models had not clearly described the mechanism of sludge compressing at very high sludge concentrations. The Vesilind equation ignores the compressional settling, usually resulting in errors to predict the sludge concentrations in full-scale secondary clarifiers. Therefore, researchers discrete the settling height into several layers with uniform concentrations in each layer, and applied first or second-order kinetics governed by solids flux theory [11,12]. However, such attempts required a substantial amount of calculation and careful parameter estimation as well. Moreover, it was difficult to directly link the model inputs and outputs to the process control of the activated sludge system.

After sludge settling, the concentrated activated sludge is recycled to the aeration tank to maintain sufficient microorganisms in the system. Determination of the return sludge concentration (*X*_r_) from a secondary clarifier is of great importance for the proper design and operation of a WWTP. By excluding the negligible concentration of outlet suspended solids, people can predict *X_r_* by mass balance calculations [13], but this method was too coarse in estimation for actual process. Later, an empirical model with many variants was proposed to calculate *X*_r_ more precisely [14]. Yet, it was not popularly used because of the sophisticated parameter estimation.

Therefore, we tried to simplify the settling models including compressional step, and investigated how to correlate the models directly to the process operations in WWTPs. In general, we used process data to regress empirical models on one easily measurable parameter and integrated with Vesilind equation to be a complete model for secondary settler. We additionally evaluated the initial settling height and slotting baffles to improve the settlers’ performance.

## 2. Materials and Methods

### 2.1. Sampling

**Figure 1:**
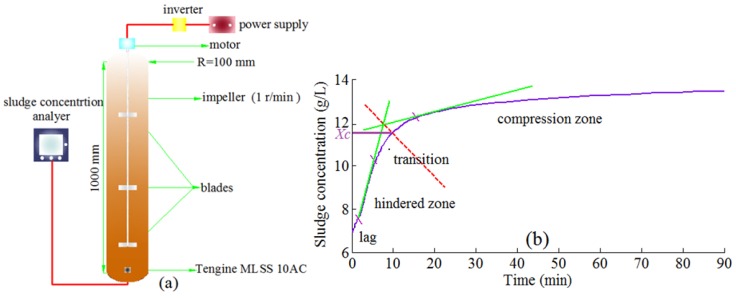
(**a**) Device used for the sludge thickening experiment (R is the radius of the settling column.); and (**b**) The activated sludge thickening process.

The experimental setup is shown in Figure 1. The height (1000 mm) and radius (100 mm) of the device were in accordance with standard methods specifications [15], and the device was equipped with a stirrer (1 r/min) driven by a speed controller engine. In addition, Tengine^®^ MLSS 10AC (Tengine, China) was used to detect the thickened sludge concentration over time at the bottom of the settling column (Figure 1a).

Activated sludge samples were taken from the diffusion aeration activated sludge systems of five different WWTPs in Beijing, China. High sludge concentrations were obtained by thickening, and low sludge concentrations were achieved by diluting the samples with sludge supernatant liquid. All activated sludge samples were analyzed within 12 h of collection with 90 min tests to minimize denitrification, especially when investigating the compression settling stage.

### 2.2. Sludge Thickening and Settling Tests

In the sludge thickening tests, the initial sludge concentration was adjusted to *X_c_* (the sludge concentration when the compression thickening commences); this was because the sludge concentration was assumed to be *X_c_* in the sludge blanket. In order to determine *X_c_*, generally a graphical method was applied, this involved using the intersection of the thickening curve and the bisector of the angle formed by the tangents to the hindered and compression zones to approximate the critical point at which the sludge thickening commences [16,17] (Figure 1b).

The initial sludge concentrations were also measured by Tengine^®^ MLSS 10AC. In addition, the *SVIs* of different WWTPs were obtained by the standard *SVI* test [18]. Five sludge settling tests were carried out at different initial sludge concentrations *X*_0_ (*i.e.*, 2.010, 3.025, 4.100, 5.180, and 6.425 g/L) with an initial sludge blanket height (*X*_0_) of 1 m within 90 min. The activated sludge sample for this test was collected from WWTP 5, with *SVI* as 230.5 mL/g.

### 2.3. Model Development

#### 2.3.1. Return Sludge Concentration Model

The experimental data for the five sludge thickening tests were divided into two groups. Data from WWTPs 1–4 were used for regression, while data from WWTP 5 were used to validation. According to the profile of sludge thickening from WWTPs 1–4, the exponential function was selected to describe the sludge concentration at the bottom of the clarifiers (*X_d_*):

(1) $$X_{d}=a{t_{s}}^{b}$$

where $t_{s}$ is the sludge thickening time, *a* and *b* are model parameters.

In the regression, the data ranges satisfied *t_s_* ≥ 0, 59.3 mL/g ≤ *SVI* ≤ 248.4 mL/g, and 7.95 g/L < *X_d_* < 14.81 g/L. In addition, by considering the turbulence by scraping or sucking of the sludge near the bottom exit of secondary settlers, we proposed the return sludge concentration (*X_r_*) as follows.

(2) $$X_{r}=kX_{d}=ka{t_{s}}^{b}$$

where *k* is a constant lower than 1. The *k* value was decided by the recycling methods, *i.e.*, *k* = 0.7 when scraping the bottom sludge and *k* = 0.5~0.7 when sucking the bottom sludge.

#### 2.3.2. Sludge Compression Settling Model

Sludge compression settling is still a difficult problem for the researchers. Hultman and Hultgren [19] proposed a dynamic model with two parameters, *i.e.*, the permeability *k* and the compression modules sludge properties *M*. Roche *et al.* [20] introduced a correcting compression parameter Ω in the Vesilind model to continuously describe the changes of velocity in the sludge thickening zone. However, the parameters *k*, *M*, and Ω in these models are difficult to measure, or impossible to be derived from process data.

We attempted to give a simple description of sludge compression settling velocity, by assuming that the sludge concentration in the compression zone is virtually homogeneous. Firstly, mass balance function was developed as Equation (3).

(3) $$x_{0}X_{0}=X_{d}x$$

where *x*_0_ is the initial sludge height and *x* is the sludge height at a certain time. In a WWTP, the parameters *x*_0_ and *X*_0_ are constants.

By combining Equations (1) and (3), we have expression for sludge height *x*.

(4) $$x=\frac{x_{0}X_{0}}{a}{t_{s}}^{-b}$$

Therefore, the sludge compression settling velocity $v_{cs}$ was developed as the first derivative of *x*.

(5) $$v_{cs}=-\frac{dx}{dt_{s}}=(\frac{bx_{0}X_{0}}{a}){t_{s}}^{-1-b}$$

By removing the settling time *t*_s_, we have *v*_cs_ in form of the concentration *X*.

(6) $$v_{cs}=-\frac{dx}{dt_{s}}=(\frac{bx_{0}X_{0}}{a}){\left(\frac{X}{a}\right)}^{\left(-\frac{1+b}{b}\right)}$$

where *v_cs_* is the sludge compression velocity, *X* is the sludge concentration, *a* and *b* are parameters.

### 2.4. Activated Sludge Fractal Dimensions

The activated sludge structure plays an important role in the settling and thickening processes, which are crucial for the overall performance and efficiency of the secondary settlers [21]. The highly irregular and disordered nature of sludge flocs has been described through the application of the theory of fractal dimensions [22]. Two- and three-dimensions ( *D_2_* and *D_3_* ) have been used frequently for the sludge flocs to study the sludge aggregates, using the Equations (7) and (8) [23,24,25].

(7) $$A\propto P^{D_{2}}$$

(8) $$V\propto P^{D_{3}}$$

where *A* is the section area of the sludge floc, *P* is the perimeter of the sludge floc, and *V* is the volume of the sludge floc, *D*_2_ and *D*_3_ are fractal indices in two- and three-dimensions, respectively. A high fractal value indicates compact sludge flocs, whereas a low value corresponds to “looser” sludge flocs [26].

Additionally, the boundary fractal dimension (*D_B_*) can be used to study the rugged boundaries of sludge flocs, which can be calculated with Equation (9) [27]. A high boundary fractal value represents a high porous configuration in the sludge flocs, whereas a low value corresponds to “denser” sludge flocs [27,28].

(9) $$A\propto P^{2/D_{B}}$$

In this study, the dimensions of the flocs were calculated by using image analysis technology [29]. The perimeter, area, and volume of the sludge floc were obtained through image Pro Plus 6.0 software (Media Cybernetics, USA). The slope of the fitting line was used as the sludge flocs’ fractal dimension.

## 3. Results and Discussion

### 3.1. Model Calibration

**Figure 2:**
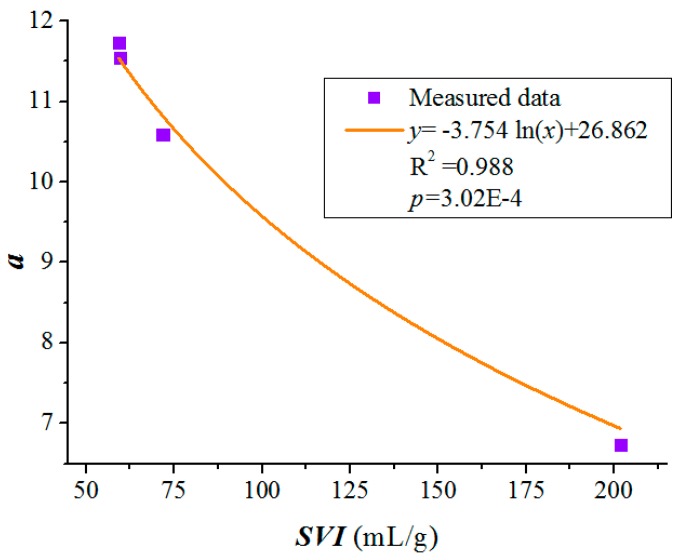
Relationship between parameter *a* and the sludge volume index (*SVI*).

By process data from WWTPs 1–4, we regressed parameters *a* in Equation (10). The *SVI*, *a*, *b*, *R*^2^, and *p*-values for all regression analyses of WWTPs 1–4 are shown in Table 1 and Figure 2. Parameter *a* was well correlated with the *SVI* value (Figure 2), and b values were more or less constant with mean value of 0.0465.

(10) $$a=-5.754\ln(SVI)+26.862\;\left(R^{2}=\;0.988,\;p=3.02\;\times \;10^{-4}\right)$$

The sensitivity of *X_d_* to *b* was $S(x,b)=\frac{\partial X_{d}}{X_{d}}\times \frac{b}{\partial b}$ < 0.2, and it could be reduced to zero quickly by decreasing *t_s_*. This indicated that even if a mean value of 0.0465 was adopted, the error of *X_d_* estimation was still very small.

Therefore, the return sludge concentration model and the sludge compression settling were obtained as:

(11) $$X_{r}=k(-5.754\ln(SVI)+26.862)\times {t_{s}}^{0.0465}$$

(12) $$v_{cs}=(\frac{0.0465x_{0}X_{0}}{-5.754\ln(SVI)+26.862}){\left(\frac{X}{-5.754\ln(SVI)+26.862}\right)}^{-22.5}$$

If the *SVI* value is measured for a WWTP, then *X_r_* and *v_cs_* can be easily determined without the need for considerable computational efforts and careful estimations of parameters.

**Table 1:** The sludge volume index (*SVI*), parameters *a* and *b*, coefficient of determination (R^2^), and *p*-values for the regression analyses of the wastewater treatment plants (WWTPs) 1–4.

WWTP	*SVI* (mL/g)	*a*	*b*	R^2^	*p*
1	59.3	11.73	0.0496	0.88	<0.001
2	59.6	11.54	0.0460	0.78	<0.001
3	71.8	10.59	0.0500	0.86	<0.001
4	201.8	6.74	0.0402	0.88	<0.001

### 3.2. Model Validation

#### 3.2.1. Return Activated Sludge Concentration Model Validation

**Figure 3:**
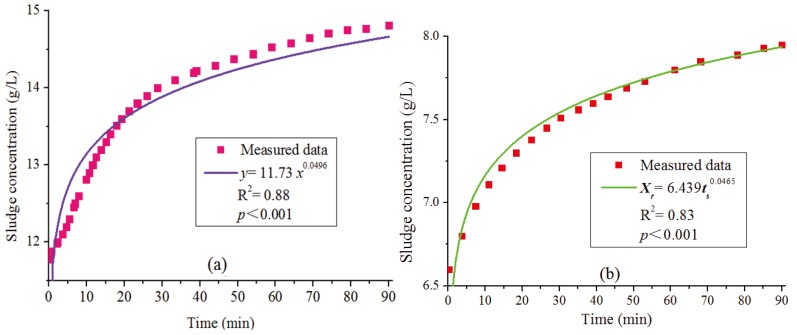
(**a**) The experimental data in wastewater treatment plant (WWTP) 1 of regression analysis applying the bottom sludge concentration model, (**b**) Proposed model validated with the measured data from wastewater treatment plant (WWTP) 5.

The regression analysis of the experimental data in WWTPs 1–4 was performed by applying Equation (1), and the results showed that the coefficient of determination (R^2^) were all close to 0.88 and the *p*-values were below 0.001. This suggests that the exponential function of Equation (1) was suitable for the sludge concentration at the bottom of the secondary clarifiers. For example, the measured data for WWTP 1 from Equation (1) had an R^2^ of 0.88 and *p*-value < 0.001 with *SVI* of 59.3 mL/g, *a* of 11.73 and *b* of 0.0496 (Figure 3a).

We applied Equation (10) to evaluate the data in WWTP 5, with the results of *SVI* value as 230.5 mL/g and parameter *a* as 6.493. The curve fitting of data in WWTP5 (Figure 3b) showed that the model evaluated the bottom sludge concentration well. The return sludge concentrations *X_r_* were monitored in range of 4.800–5.800 g/L in WWTP 5, and the calculated values of *X_r_* were in range of 4.620–5.635 with *k* = 0.7. This result proved that the return sludge concentrations were well predicted.

#### 3.2.2. New Sludge Compression Model Validation

In WWTP 5, the sludge compression settling model could be obtained from Equation (12) with an *x*_0_ of 1 m and an *X*_0_ of 6.600 g/L. Thus,

(13) $$v_{cs}=0.477\times {\left(\frac{6.439}{X}\right)}^{22.5}$$

**Figure 4:**
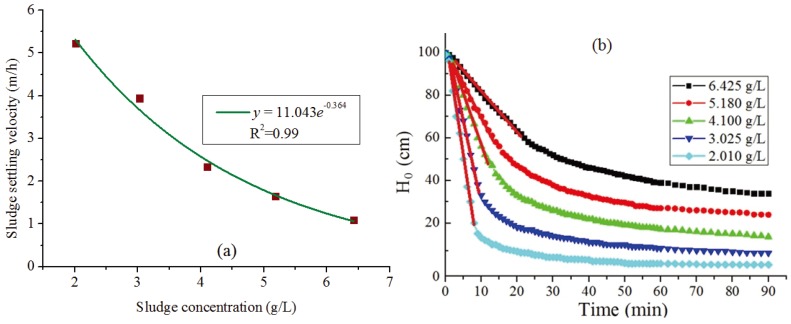
(**a**) Batch settling curves in wastewater treatment plant (WWTP) 5 (slope of the bold red line is the sludge hindered settling velocity at different initial sludge concentrations), (**b**) Relationship between the sludge hindered settling velocity and the corresponding initial sludge concentration.

Additionally, the sludge hindered settling velocity (Figure 4a) could be estimated by the data of sludge batch settling tests (Figure 4b), by using the Vesilind Equation [10]:

(14) $$v_{s}=11.043e^{-0.364X}$$

This allowed for the development of the integral sludge settling model (including both the sludge hindered settling and compression settling), as shown below:

(15) $$v=\{\begin{array}{c} v_{0}e^{-nx},X\leq X_{c}\\ (\frac{0.0465x_{0}X_{0}}{-5.754\ln(SVI)+26.862}){\left(\frac{X}{-5.754\ln(SVI)+26.862}\right)}^{-22.5},X\geq X_{c} \end{array}$$

when the sludge entered the compression zone, the settling velocity (*v_cs_*) is obviously lower than the hindered settling velocity (*v_s_*). Therefore, if the solids flux theory contains the sludge hindered settling model only, the predicted sludge concentrations would be larger than the true values. Ekama *et al.* stated that the application of the solids flux procedure over-estimated the permissible solids loading by around 25% [30], which supported our model’s performance.

### 3.3. Factors Affecting the Sludge Thickening

The factors such as the initial sludge height (*x*_0_) and stirring rates were analyzed, given their potential to influence sludge thickening and affect the operation of WWTPs.

#### 3.3.1. Sludge Height

**Figure 5:**
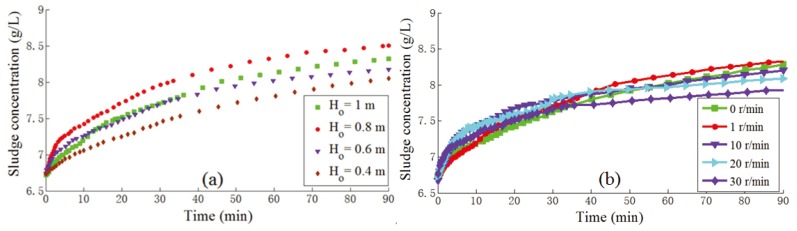
Sludge concentration at the bottom of secondary settlers under (**a**) different initial sludge heights and (**b**) different stirrer speeds.

The sludge height is an important operational factor for optimizing the secondary settlers operation. A higher sludge height could produce a larger return sludge concentration. We analyzed the bottom sludge concentration in different initial sludge heights. The bottom sludge concentration was 8.500 g/L at an *x*_0_ of 1 m, but was only 8.050 g/L at an *x*_0_ of 0.4 m (Figure 5a). The sludge was collected from WWTP 5 with an experimental *X*_0_ of 6.600 g/L.

This indicated that higher secondary settlers could cause higher hydraulic liquid pressure in the lower section, which may then contribute to sludge thickening and inhibit denitrification. Therefore, the height of secondary settlers can be increased in the WWTPs to produce a higher return sludge concentration, thus ensuring adequate microbial activity in the aeration tank. Meanwhile, a higher height of secondary settlers could contribute to the production of better quality water from a WWTP. Therefore, increasing the height of secondary settlers should provide multiple benefits in WWTPs.

#### 3.3.2. Stirring Rates

The fluid characteristics, such as flocculation and compressibility, play an important role in the sludge thickening zone, which is easily influenced by the turbulence. Generally, the stirrer was used to simulate turbulence in secondary settlers. With the sludge concentration increasing, it becomes more difficult for the sludge thickening because of the larger drag force. We conducted batch experiments to record bottom sludge concentrations at different stirring rates.

Figure 5b shows that stirring rates about 1 r/min were helpful for sludge thickening process. Slight stirring generated proper drag forces to enhance the flocculation and thus performed better than the control test (without stirring). However, when the stirring rates increased to 10–30 r/min, the sludge thickening became worse because the drastic turbulence affected the transition and compression of the sludge thickening seriously. In general, the turbulence in the secondary clarifiers was very strong due to the incautious geometric design and equipment selection for the settler. Therefore, some methods like slotted baffles in the inlet of the secondary settlers should be used to control the adverse effects.

### 3.4. Activated Sludge Fractal Dimension

**Figure 6:**
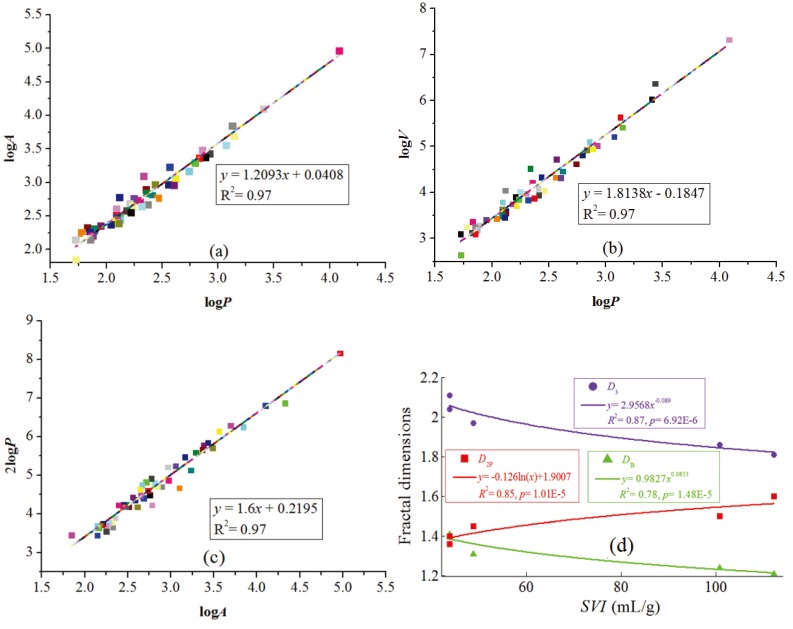
(**a**–**c**) The *D*_2_, *D*_3_, and *D_B_* for the sludge flocs from wastewater treatment plant (WWTP) 5, (**d**) The *D*_2_, *D*_3_, and *D_B_* for the sludge flocs from WWTPs 1–5.

The capability of sludge thickening could be expressed by *SVI*, for which lower value clues a better thickening performance. Furthermore, the capacity were determined by the sludge shapes and density. The fractal number *D*_2_, *D*_3_, and *D_B_* values for the sludge flocs in WWTP 5 were 1.2, 1.8, and 1.6, respectively, as shown in Figure 6a–c. By fitting all data from fractal number for WWTPs 1–5 (Figure 6d), we tried to link the *SVI* with the fractal properties of sludge. *D*_2_ and *D*_3_ for the sludge flocs decreased when the *SVI* values increased, implying that the sludge flocs with more compact structure had lower *SVI* values and thus showed better settling performance. However, the *D_B_* for the sludge flocs increased along with *SVI*, showing that the lower *SVI* sludge flocs had a less porous structure. As a result, the compact and little porous structures of sludge flocs can contribute to their good thickening capability.

## 4. Conclusions

We developed a simple empirical model with easily measurable parameters (*t_s_* and *SVI*) for sludge settling process, without intensive calculations or parameter estimation. With the help of this model, the operators can determine the return sludge concentration with *SVI* and change the sludge thickening time *t_s_* in the second clarifier to optimize *X_r_*. Meanwhile, a sludge settling model was also developed (including for both sludge hindered and compression settling), which could improve the solids flux theory. The fractal number *D*_2_, *D*_3_, and *D_B_* for the sludge flocs were well correlated with the *SVI* values, and the results showed that the sludge with low *SVI* values possessed a “tighter” and “denser” structure, thus leading to higher settling and thickening velocity. Additionally, by increasing the height of the secondary settlers and using slotted baffles in the inlet, the performance of secondary clarifiers could be improved.
